# Ulceration of the Cœcum,—Hæmorrhage,—Death

**Published:** 1850-02

**Authors:** H. A. Ramsay

**Affiliations:** Raysville, Georgia


*Ulceration of the Cœcum,—Hæmorrhage,—Death.* By H. A. Ramsay, M. D., Raysville, Georgia.

On the 1st ult. I was called to see Emanuel, belonging to Mr. C——, of this vicinity. The patient was a stout, robust man, ætat. 24. He had been complaining of some slight pain in his *bowels* and *lumbar* portion of the spine for several weeks, together with some perversion of his appetite; not sufficient, however, to prevent him from being about, and attending to light work. On the 1st he grew suddenly worse, and was obliged to go to his bed. When sent for I found him rather restless, pulse soft and voluminous, but not increased in action; skin warm and comfortable, with the exception of his hands, which were cool; tongue soft, sides white, middle red; pressure upon the epigastric region and the abdomen generally produced some evidence of tenderness; he said, however, that he felt no pain, yet at the same time he was giving vent to constant groans and sighs. I was induced to think he certainly *felt* pain, from his complaining so constantly, but he positively denied the existence of any, although repeatedly interrogated by myself and Dr. Collins, who saw him subsequently. The patient said he had had regular alvine evacuations daily; he was rational, and without mental disturbance at any time; his spine exhibited no tenderness on pressure; his stomach was not disturbed by *nausea*, nor was his urinary secretion in the least deranged. This is all the information that could be elicited by Dr. Collins and myself, although we examined him very minutely. We prescribed calomel in repeated doses during the night, sinapisms, and an opiate to calm his restlessness, and emollient poultices to the abdomen. At 2 o'clock at night the mercurial produced a green biliary evacuation, shortly after which he had a copious discharge of clotted blood; but few minutes had elapsed ere another took place. These continued to increase greatly, and alarmed those watching. I was called immediately, and found the man weltering in his blood, *pulseless*, rolling from side to side of his bed, *moaning* but *rational*, and declaring that he felt no pain. I examined the rectum and found the blood running almost in a stream. Seeing he would die in a few moments, unless relieved, I plugged the *rectum*, gave him freely of brandy, applied mustard plasters, and ordered a laudanum enema—having despatched a boy for a syringe, but ere he returned the patient had sunk.

Two hours after death, in the presence of Dr. Collins and several gentlemen, I made an autopsy. The stomach exhibited no disease. The spleen was corrugated. The liver contained some dark grumous blood, but looked natural and healthy. The intestinal tube did not exhibit any marked signs of disease, excepting in the cœcum, where it was ulcerated for nearly two inches, the ulceration presenting a dark greenish color, like mouldy cheese; near the middle of the ulcer there was exposed the mouth of an artery, the coats of which had evidently been destroyed, thus producing the hæmorrhage. The calibre of the bowel was very much diminished; the appendix vermiformis was smooth and indurated; and about the point of ulceration the mesentery presented some marks of purulent deposition. We did not examine the brain; the bladder was sound; there was some effusion into the pericardium; the lungs were healthy.

The patient died in less than three hours from the time the hæmorrhage began.

How long could this ulcer have existed before his death?

